# Effects of isoleucine 135 side chain length on the cofactor donor-acceptor distance within F_420_H_2_:NADP^+^ oxidoreductase: A kinetic analysis^★^

**DOI:** 10.1016/j.bbrep.2016.11.012

**Published:** 2016-11-30

**Authors:** Cuong Quang Le, Mercy Oyugi, Ebenezer Joseph, Toan Nguyen, Md Hasmat Ullah, Joshua Aubert, Thien Phan, Joseph Tran, Kayunta Johnson-Winters

**Affiliations:** Department of Chemistry and Biochemistry, University of Texas at Arlington, TX 76019-0065, USA

**Keywords:** Fno, F_420_H_2_:NADP^+^, oxidoreductase, *wt*Fno, wild-type Fno, NADP^+^, nicotinamide adenine dinucleotide phosphate, F_420_ cofactor, 7,8-didemethyl-8-hydroxy-5-deazariboflavin-5′-phosphoryllactyl(glutamyl)_n_glutamate, *A. fulgidus*, *Archaeoglobus fulgidus*, *E. coli,*, *Escherichia coli*, IPTG, isopropyl β-D-1-thiogalactopyranoside, FO, precursor of F_420_ cofactor, LB, Luria Bertani broth, *K*_d_*,*, dissociation constant, *K*_m_, Michaelis-Menten constant, *k*, rate constant, *k*_cat_, catalytic rate constant (turnover number), *k*_cat_ /*K*_m_, catalytic efficiency, I135, Isoleucine 135, PEI, Polyethyleneimine, F_420_H_2_: NADP^+^ oxidoreductase, NADP, F_420_ cofactor, Steady-state kinetics, Pre steady-state kinetics, Dissociation constants, Negative cooperativity, Half-site reactivity

## Abstract

F_420_H_2_:NADP^+^ Oxidoreductase (Fno) catalyzes the reversible reduction of NADP^+^ to NADPH by transferring a hydride from the reduced F_420_ cofactor. Here, we have employed binding studies, steady-state and pre steady-state kinetic methods upon *wt*Fno and isoleucine 135 (I135) Fno variants in order to study the effects of side chain length on the donor-acceptor distance between NADP^+^ and the F_420_ precursor, FO. The conserved I135 residue of Fno was converted to a valine, alanine and glycine, thereby shortening the side chain length. The steady-state kinetic analysis of *wt*Fno and the variants showed classic Michaelis-Menten kinetics with varying FO concentrations. The data revealed a decreased *k*_cat_ as side chain length decreased, with varying FO concentrations. The steady-state plots revealed non-Michaelis-Menten kinetic behavior when NADPH was varied. The double reciprocal plot of the varying NADPH concentrations displays a downward concave shape, while the NADPH binding curves gave Hill coefficients of less than 1. These data suggest that negative cooperativity occurs between the two identical monomers. The pre steady-state Abs_420_ versus time trace revealed biphasic kinetics, with a fast phase (hydride transfer) and a slow phase. The fast phase displayed an increased rate constant as side chain length decreased. The rate constant for the second phase, remained ~2 s^−1^ for each variant. Our data suggest that I135 plays a key role in sustaining the donor-acceptor distance between the two cofactors, thereby regulating the rate at which the hydride is transferred from FOH_2_ to NADP^+^. Therefore, Fno is a dynamic enzyme that regulates NADPH production.

## Graphical abstract

**Figure:**
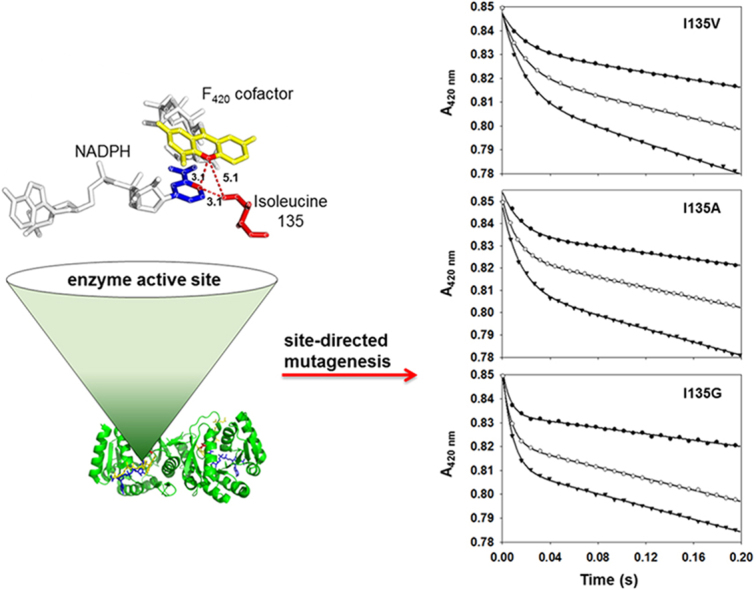

## 1 Introduction

**Fig. 1:**
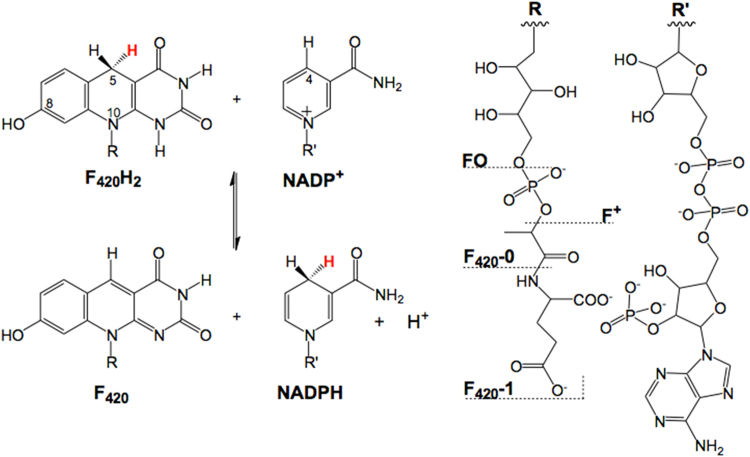
**Left side:** Fno catalyzed reaction. Fno catalyzes the reversible reduction of NADP^+^. The pro-*S* hydride on carbon 5 of F_420_H_2_ (shown in red) is transferred to carbon 4 of NADP^+^, producing NADPH. **Right side:** R represents the side chain of the F_420_ cofactor and its precursors, which are structurally separated by the dashed lines. The structural side chain for FO includes the ribitol moiety of the R group. The structural side chain for F^+^, includes the ribitol and the phosphate moieties of the R group. The structural side chain for F_420_-0, includes the ribitol, phosphate and lactyl moieties of the R group. Finally, the F_420_-1 structural motif includes the entire R-side chain (the number 1 represents the length of the polyglutamate tail) [1], [4], [5], [8]. R′ represents the NADPH side chain. (For interpretation of the references to color in this figure, the reader is referred to the web version of this article.)

F_420_H_2_:NADP^+^ Oxidoreductase (Fno) catalyzes the reversible reduction of NADP^+^ to NADPH by transferring a hydride from carbon 5 on the *pro-S* side of the F_420_ cofactor (7,8-didemethyl-8-hydroxy-5-deazariboflavin-5′-phosphoryllactyl(glutamyl)_n_glutamate)) to carbon 4 on the *pro-S* side of NADP^+^(Fig. 1). Fno has been purified from the sulfate reducing archaea, *Archeoglobus fulgidus*[1] as well as from several methanogenic organisms [2], [3], [4], [5]. Fno has also been isolated from bacteria such as *Halobacterium cutirubrum*[6] and *Streptomyces griseus*[7]. We have modified the expression and purification protocol of Fno from *A. fulgidus* to eliminate nucleic acid contamination, as reported elsewhere [8].

Equilibrium studies with Fno from *Methanobacterium thermoautotrophicum* have revealed that the reduction potential of FO (F_420_ cofactor precursor, Fig. 1) is between −340 and −350 mV [6]. The ionization of the 8-OH substituent (p*K*_a_ of 5.7) in the oxidized FO, stabilized by cross-conjugation throughout the tricyclic deazaflavin, suppresses FO reactivity toward redox chemistry. However, in the reduced FO (FOH_2_), the phenolic group is electronically isolated to a single benzoic ring, resulting in a higher p*K*_a_ of 9.7 [6]. The present experiments were conducted with our synthesized F_420_ cofactor precursor, FO, because the F_420_ cofactor is difficult to isolate in sufficient quantity for biophysical studies (Fig. 1) [8]. Synthetic FO is fully catalytically active with Fno and has a higher purity than what can be produced from microorganisms. We have reported a detailed description of the improved synthesis of FO elsewhere [9].

**Fig. 2:**
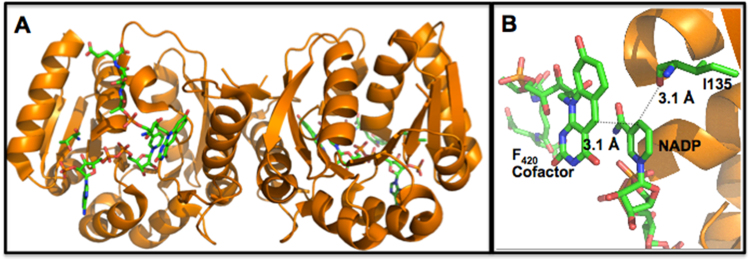
Crystal structure of Fno. **A:** homodimeric quaternary structure of Fno, in the presence of oxidized F_420_ cofactor and NADP^+^. **B:** active site of Fno, PDB file 1jax [1]. The C5 of F_420_ and C4 of NADP^+^ are 3.1 Å apart, positioned for a direct hydride transfer. I135 is positioned on the NADP^+^ side, with a 3.1 Å distance from NADP^+^ within the crystal structure.

The crystal structure of Fno from *A. fulgidus* was solved in 2001 (Fig. 2) [1]. According to the structural analysis, Fno is a homodimer with an α,β twisted fold motif. Fno has one F_420_ cofactor molecule and one NADP^+^ molecule bound per monomer (Fig. 2A). The C5 of F_420_ and C4 of NADPH are within 3.1 Å of one another (Fig. 2B), which is an acceptable donor-acceptor distance for a hydride transfer between these two atoms [1], [6], [10], [11], [12], [13]. Previous steady-state kinetic analysis confirmed the existence of the ternary complex with *K*_m_ values of 20 µM and 40 µM for F_420_H_2_ and NADP^+^, respectively, at 65 °C and pH 5.5 [1], [2], [3], [4], [5], [7], [14], [15]. While the order of substrate addition could not be determined, the steady-state kinetic data and additional experiments suggest that the presence of NADP^+^ aids F_420_ binding [1].

Our initial kinetic studies with *wt*Fno included a steady-state kinetic analysis using FO (Fig. 1) [9]. The steady-state kinetic analysis of *wt*Fno at 22 °C followed a typical hyperbolic curve with varying FO concentrations. The kinetic parameters, *K*_m_ and *k*_cat,_ were determined by fitting the plot of rate constant *k* (s^−1^) versus varying FO concentration (μM) to the Michaelis-Menten hyperbolic equation. The *K*_m_ and *k*_cat_ determined from the plot were 4.00±0.39 μM and 5.27±0.14 s^−1^, respectively [9]. Our steady-state kinetic analysis of Fno with varying concentrations of NADPH did not follow typical Michaelis-Menten kinetics at NADPH concentrations higher than 100 μM up to 1.7 mM. When these data were converted to a double reciprocal plot, it displayed a downward concave shape, which is indicative of negative cooperativity [17]. The pre steady-state data showed biphasic kinetics, with a burst phase and a slow phase. When varying Fno concentrations, the amplitude of the burst phase corresponded to only ~50% FO reduction. This behavior suggests half-site reactivity [16]. Half-site reactivity has not been seen previously with Fno, or other F_420_ cofactor dependent enzymes, thus providing potential valuable new insights into enzymes that use this unique cofactor.

**Fig. 3:**
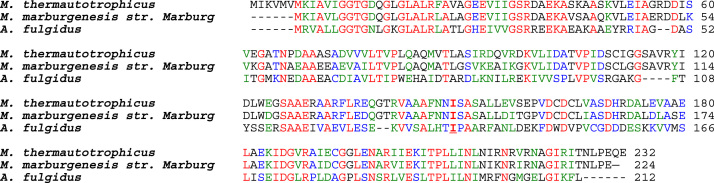
Sequence alignment of Fno from various sources. Conserved amino acids are shown in red, green are strongly similar amino acids, blue are weakly similar, while black are not conserved. Note: I135 (shown in bold) is conserved. The online program, Clustal ω was utilized to create the amino acid sequence alignment presented here. (For interpretation of the references to color in this figure, the reader is referred to the web version of this article.)

Isoleucine is a conserved amino acid amongst many enzymes that utilize NADPH. Within Fno, the residue numbering for the conserved active site isoleucine (I) is I135 (Fig. 2, Fig. 3). The distance between the carbonyl oxygen of I135 and atom C4 of NADP^+^ is 3.1 Å (Fig. 2b) [1]. Warkentin et. al suggest that this distance is likely of catalytic relevance [1]. In this study, I135 was systematically reduced by one carbon for each variant (I135V, I135A, and I135G, respectively), in order to investigate the effects of the I135 side chain length on the donor-acceptor distance between the two cofactors. These variants were examined through binding studies, steady-state and pre steady-state kinetic methods. The data displayed negative cooperativity kinetics and half site reactivity. Additionally, we found that as the side chain length decreased, there was an increase in the hydride transfer rate constant. Our data suggests that I135 aids in sustaining the donor-acceptor distance between the two cofactors, thereby regulating the rate of NADPH production within the cell.

## 2 Materials and methods

### 2.1 Reagents

FO synthesis was reported elsewhere [9]. NADPH was purchased from Akron Biotech. The pET24b plasmid used for Fno gene insertion and mutagenesis was purchased from Novagen. Luria Broth was purchased from US Biologicals. Isopropyl β-D-1-thiogalactopyranoside (IPTG) was purchased from Gold Biotechnology. MES buffer was purchased from Acros Organics.

### 2.2 Mutagenesis

Cloning of the *Fno* gene into the pET24b plasmid was conducted by the company Genescript. Site-directed mutations were generated in pET24b using QuikChange site directed mutagenesis (Agilent), according to the manufacturer's protocol. I135 Fno primers for mutagenesis studies are shown in Table S1 of Supporting Information.

### 2.3 Expression and purification

The mutated Fno plasmids were transformed into C41(*DE3*) *E. coli* cells according to the Lucigen Technologies protocol and then used for Fno expression. A detailed report of the expression and purification protocol has been reported by Le et. al. [8]. Briefly, Fno was purified using heat precipitation at 90 °C for 30 min, followed by two ammonium sulfate fractionation steps (0–40% and then 40–70%) at 4 °C. Next, polyethyleneimine (PEI) precipitation, and anion exchange chromatography, using a DEAE-Cellulose column (Sigma) were employed for further purification. Finally, an S-200 Sephacryl HR Size Exclusion column (GE Healthcare) was utilized to desalt the Fno sample [8].

### 2.4 Binding of FO and NADPH to Fno variants

The FO and NADPH binding experiments have been reported previously for *wt*Fno [16]. The binding experiments for the Fno variants were conducted in a similar manner as *wt*Fno for comparison and are described here. The experiments were performed using a Horiba FluoroMax Spectrofluorometer. Individual binding assays were monitored in a 160 μl Spectrosil® Quartz sub-micro cell from Starna Cells. Each sample was excited at 290 nm and the emission scans were monitored between 300–800 nm. The excitation and emission slit widths were 4 and 8 nm, respectively. To obtain the dissociation constant (*K*_d_) of FO, either 1 or 2 μl aliquots of FO (0–300 nM) were titrated into a solution containing 0.2 μM Fno in 50 mM MES/NaOH at pH 6.5.

To obtain the *K*_d_ of NADPH, varying concentrations of NADPH (0–1780 μM) were titrated with 0.2 μM of Fno in 50 mM MES/NaOH at pH 6.5. The binding assay was monitored as described above for the FO binding studies. A decrease in tryptophan emission at 340 nm was observed for both FO and NADPH titrations and used for calculation of the dissociation constants [16], [17]. A plot of change in fluorescence *vs*. substrate concentration was used to determine the dissociation constants (Fig. S1).

To determine the Hill coefficient and the dissociation constants for both FO and NADPH binding data, the plots were fitted to a sigmoidal function (Eq. (1)) using Sigma Plot version 13.0.

(1) $$\Delta F=\left[F_{\max}{\left[L\right]}^{n}/(K_{d}+{\left[L\right]}^{n})\right]$$

where, ΔF is the change in fluorescence emission at 340 nm caused upon addition of either FO or NADPH as the ligand (L). F_max_ is the maximum normalized fluorescence (F_max_ =1). Then, each normalized data point was divided by the F_max_ to obtain the fractional saturation point. *K*_d_ is the dissociation constant and *n* is the Hill coefficient. The ligand concentration was corrected for dilution during addition to the assay.

### 2.5 Steady-state kinetics of Fno variants

The steady-state kinetic measurements were obtained using a Hitech Scientific DX2 stopped-flow spectrophotometer to capture more of the initial rate for analysis. This is consistent with our previous steady-state kinetic studies of *wt*Fno. To obtain the FO steady-state kinetic parameters, a sample of 0.2 μΜ Fno and 600 µΜ ΝΑDPH in 50 mM MES/NaOH, pH 6.5 was mixed with varying FO concentrations (1.3 μM to 30 μM in 50 mM MES/NaOH, pH 6.5) at 22 °C. FO reduction was monitored at 420 nm. The individual rate constants, *k*, were obtained by dividing the initial rates by the Fno concentration. The plots of *k* versus FO concentrations were fitted to the Michaelis-Menten equation (Eq. (2)), where *k* is the initial rate constant, *k*_cat_ is the turnover number, *K*_m_ is the Michaelis-Menten constant, and [S] is the substrate concentration (Fig. S2).

(2) $$k=k_{cat}\left[S\right]/\left(K_{m}+\left[S\right]\right)$$

To obtain the NADPH steady-state kinetic parameters, a sample of 0.2 μΜ Fno and 25 µΜ FO in 50 mM MES/NaOH, pH 6.5 was mixed with varying NADPH concentrations (~2–1700 µM in 50 mM MES/NaOH, pH 6.5). NADPH and FO concentrations were determined using an extinction coefficient of 6.22 mM^−1^cm^−1^ at 340 nm (in 50 mM Tris-HCl, pH 7.4) and 41.4 mM^−1^cm^−1^ at 420 nm (in 50 mM potassium phosphate buffer, pH 7.0), respectively [3]. The extinction coefficient of F_420_ and FO cofactor are pH dependent. The appropriate extinction coefficient used to calculate the initial rates of the Fno reaction in the presence of FO at pH 6.5 and a wavelength of 420 nm is 34.7 mM^−1^cm^−^^1^. The assay was conducted at pH 6.5 to shift the absorbance peak of FO from 400 to 420 nm in order to avoid any potential interference from NADPH.

**Fig. 4:**
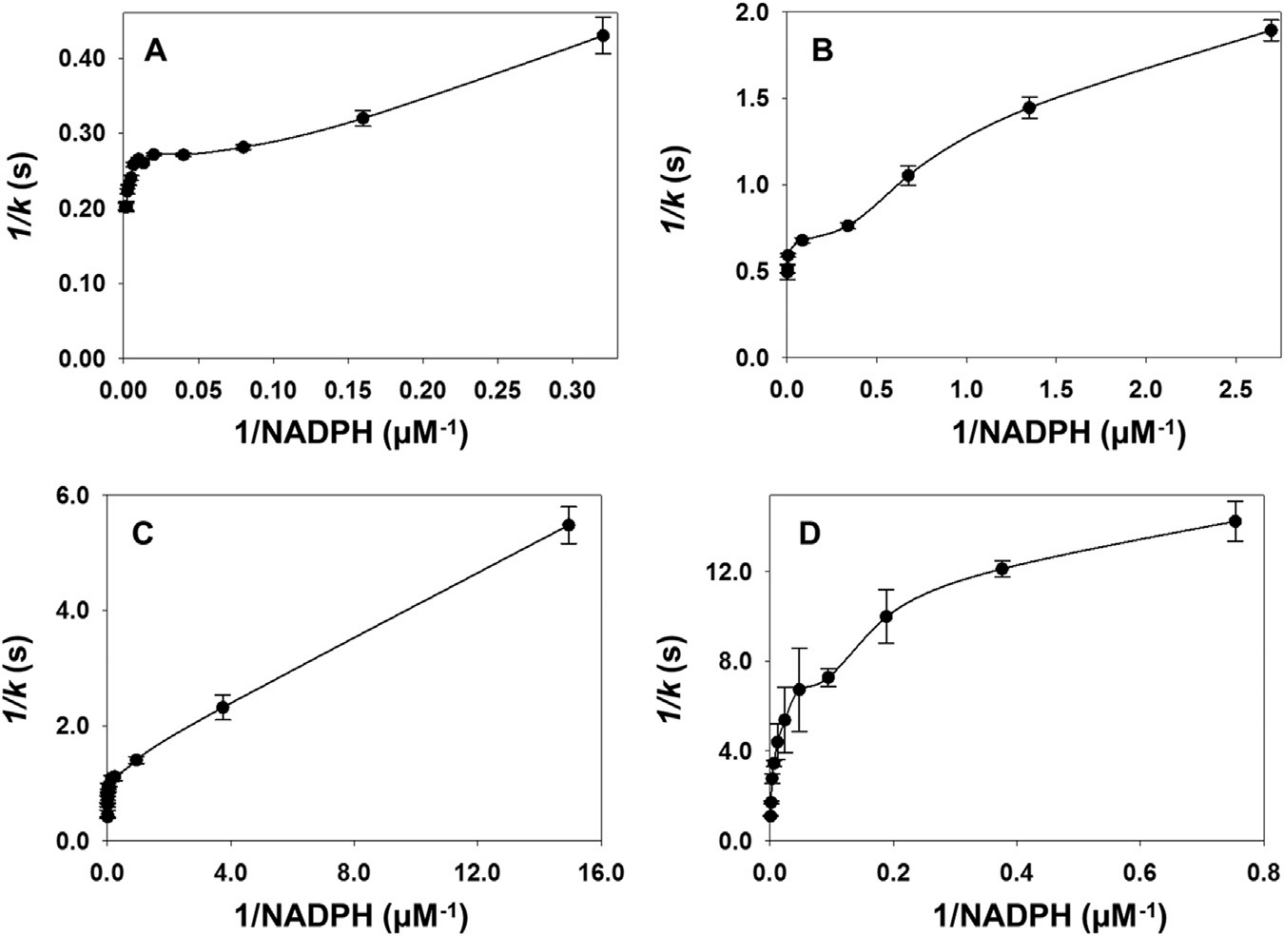
The steady state double-reciprocal plots for *wt*Fno (A)**,** I135V Fno (B)**,** I135A Fno (C) and I135G Fno (D) by varying NADPH concentrations. The reaction is carried out with 25 µM FO and 0.2  μM Fno in 50 mM MES/NaOH (pH 6.5) buffer at 22 °C. These plots were made by plotting 1/*k vs.* 1/[NADPH], displaying a concave downward curvature, which indicates negative cooperativity [17].

The steady-state plot of *k* versus NADPH concentrations did not fit to the typical Michaelis-Menten equation (Eq. (2)) because the data were biphasic. Therefore, SigmaPlot version 13.0 was used to fit the NADPH data to Eq. (3) (see Fig. S3) [18].

(3) $$k=\left(k_{cat1}\left[S\right]\right)/\left(K_{m1}+\left[S\right]\right)+k_{cat2}\left[S\right]/\left(K_{m2}+\left[S\right]\right)$$

where, *k* is the first order macroscopic rate constant and *K*_m1_ and *K*_m2_ are the Michaelis-Menten constants of the first and second phases of the plot, respectively. *k*_cat1_ and *k*_cat2_ are the catalytic rate constants of the first and second phases of the plot, respectively, and [S] is NADPH concentration. A detailed calculation of the NADPH parameters can be found in the supplemental information. The plots of 1/*k* versus 1/[NADPH] were also made (Fig. 4) and fitted to the double reciprocal equation (Eq. (4)) for each of the two phases (Fig. 4), where *k* is the first-order rate constant, *K*_m_ is the Michaelis-Menten constant, *k*_cat_ is the turnover number, and [S] is NADPH concentration.

(4) $$1/k=\left(K_{m}/k_{cat}\left[S\right]\right)+\left(1/k_{cat}\right)$$

### 2.6 Pre steady-state kinetics of Fno variants

The rapid kinetic experiments of Fno variants were conducted in a similar manner as our previous studies of *wt*Fno [16]. These experiments were performed using a Hitech Scientific DX2 stopped-flow spectrophotometer at 22 °C. Under multiple turnover conditions, a sample of 1.0 µM Fno and 10 µM NADPH in 50 mM MES/NaOH at pH 6.5 was mixed against 25 µM FO in 50 mM MES/NaOH at pH 6.5, using the diode-array mode between 350–800 nm. However, FO reduction was followed at 420 nm. The multiple turnover experiments were repeated using 1.5 µM Fno and then a third time with 2.0 µM Fno for each of the three Fno variants.

**Fig. 5:**
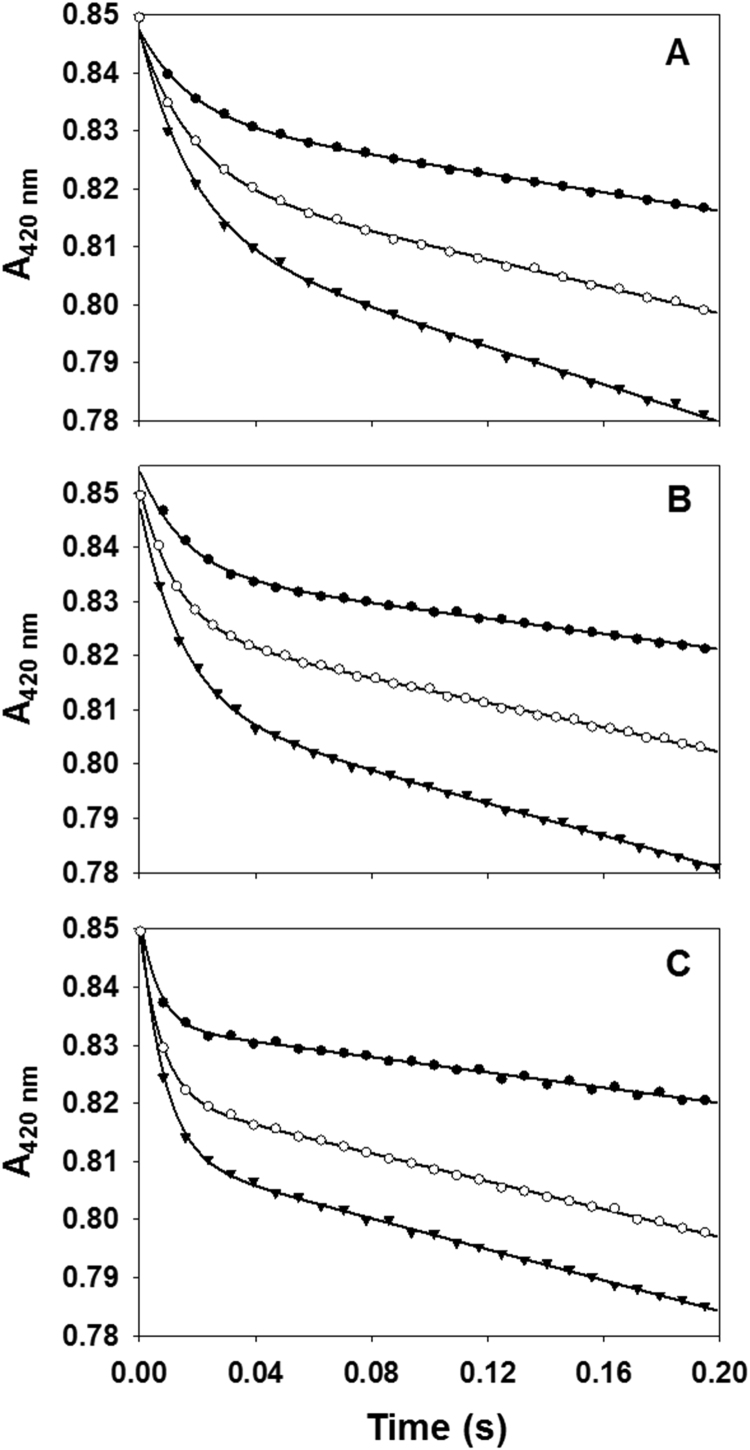
The Fno pre steady-state traces at 420 nm. The hydride is transferred to FO from NADPH by Fno. Each trace represents varying Fno concentrations: 1.0 μM Fno (solid circles), 1.5 μM Fno (open circles), and 2.0 μM Fno (solid triangles). The plots were fitted to Eq. (5) and represent the three Fno variants as follows: **A** (I135V Fno), **B** (I135A Fno), and **C** (I135G Fno). The reactions were carried out in 50 mM MES/NaOH (pH 6.5) buffer at 22 °C. Fno was mixed with 10 μM NADPH, forming the Fno-NADPH complex. FO (25 μM) in 50 mM MES/NaOH, pH 6.5 was then mixed with the Fno-NADPH complex. The detailed calculation of kinetic parameters and plot fitting of these graphs along with *wt*Fno graphs are shown in the supplemental information.

The absorbance at 420 nm *vs.* time plots were fitted to the exponential decay equation (Eq. (5)) using SigmaPlot version 13.0 (Fig. 5).

(5) $$Absorbance\;=A_{0}e^{\left(-kt\right)}-\left(vt\right)+c$$

where, A_0_ is the amplitude of the burst phase, t is time in seconds, *k* is the observed burst rate constant, *v* is the slow phase rate from which the slow phase rate constant is calculated (see supplemental information). We obtained the slow-phase rate constant (*k*_s_) by dividing the slow- phase rate (*v*_s_) by the total Fno concentration in the reaction sample, after converting *v*_s_ into the unit of µM s^−1^ using the FO extinction coefficient. In Eq. (5), c accounts for the non-zero baseline. A detailed calculation of the pre steady-state kinetics of Fno variants can be found in the supplemental information and the values are reported in Table 4. It is important to note that our stopped-flow experiments did not require anaerobic conditions. The re-oxidation of the FO cofactor with atmospheric oxygen was not observed in the time period of the pre steady-state conditions.

## 3 Results

### 3.1 Binding studies of I135 Fno variants

The *K*_d_ values for the Fno variants (I135A, I135V and I135G) with FO and NADPH were obtained using the conditions previously reported for *wt*Fno (see Table 1 and Supporting information Fig. S1) [16]. Table 1 displays the *K*_d_ values for *wt*Fno and Fno variants. These values are within the nM range and therefore indicate that Fno has a high affinity for both FO and NADPH. With respect to FO, the *K*_d_ values for the Fno variants are greater than that of *wt*Fno. The data suggest that all three Fno variants have lost some affinity for FO. Like FO, the variants display decreased affinity for NADPH, with the exception of I135G Fno. The Hill coefficients for *wt*Fno and the Fno variants (I135A, I135V and I135G) are all less than 1 (Table 1).

**Table 1:** Dissociation constants and Hill coefficients of FO and NADPH for *wt*Fno and I135 variants. The binding studies were carried out in 50 mM MES/NaOH buffer (pH 6.50) at 22 °C in a Horiba FluoroMax Spectrofluorometer. FO or NADPH was titrated into 0.2 µM Fno and the fluorescence emission was monitored at 340 nm after excitation at 290 nm.

**Fno**	***K***_**d**_^**FO**^**(nM)**	***K***_**d**_^**NADPH**^**(nM)**	***n***^**FO**^	***n***^**NADPH**^
*wt*Fno	3.6±0.7^a^	2.0±0.3^a^	0.57±0.06^a^	0.61±0.03^a^
I135V	7.5±0.9	7.4±1.1	0.64±0.04	0.81±0.09
I135A	5.6±0.2	6.7±0.7	0.79±0.03	0.82±0.07
I135G	6.9±0.3	1.5±0.1	0.80±0.03	0.80±0.03

a values obtained from reference 16.

### 3.2 Steady-state kinetics of I135 Fno variants

The steady-state plots of *k vs*. FO concentration for the Fno variants displayed typical Michaelis-Menten kinetics as previously seen with *wt*Fno [9]. The kinetic parameters *K*_m_ and *k*_cat_ of the Fno I135 variants were obtained by fitting the plots to the Michaelis-Menten equation (Eq. (2), Fig. S2). The catalytic efficiency, *k*_cat_*/ K*_m_ was obtained by dividing *k*_cat_ by the *K*_m_. The data showed that as the length of the side chain decreased, there was a decrease in *k*_cat_ and catalytic efficiency with no significant effect on the *K*_m_ (Table 2).

**Table 2:** FO steady-state kinetics parameters for *wt*Fno and I135 variants. The steady-state kinetic measurements were carried out using a Hitech Scientific DX2 stopped-flow spectrophotometer at 22 °C. A solution of 0.2 μΜ Fno and 600 μM NADPH in 50 mM MES/NaOH at pH 6.5 was mixed with varying FO concentrations (1.3 μM to 30 μM).

**Fno**	***k***_**cat**_***(*****s**^**−1**^***)***	***K***_**m**_^**FO**^**(µM)**	***k***_**cat**_***/K***_**m**_***(*****M**^**−1**^**s**^**−1**^***)***
*wt*Fno	5.3±0.1^a^	4.0±0.4^a^	1.3×10^6^±1.4×10^5^^a^
I135V	1.8 0.1	3.7± 0.4	4.9×10^5^±5.5×10^4^
I135A	1.6±0.1	3.6±0.5	4.5×10^5^±6.6×10^4^
I135G	0.7±0.0	3.6±0.4	2.0×10^5^±2.310^4^

a Values obtained from reference 16.

As seen for the *wt*Fno [16], the plot of *k* versus NADPH concentration did not display the typical hyperbola at NADPH concentrations greater than 100 µM (Fig. S3). The plots consisted of two phases and displayed non Michaelis-Menten behavior with increasing concentrations of NADPH (Fig. S3). A detailed calculation of the NADPH steady-state kinetics parameters can be found in the supplemental information and the values obtained are reported in Table 3. The data revealed that as the length of the side chain decreased, there was a decrease in *k* for the first phase and a decrease in *k*_cat_ for the second phase. The data also revealed a decrease in *K*_m_ for both phases, except I135G Fno.

**Table 3:** NADPH steady-state kinetics parameters for *wt*Fno and I135 variants. The steady-state kinetic measurements were carried out using a Hitech Scientific DX2 stopped-flow spectrophotometer at 22 °C. A solution of 0.2 μΜ Fno and 25 μM FO in 50 mM MES/NaOH at pH 6.5 was mixed with varying NADPH concentrations (2 μM to 1700 μM).

**Fno**	**Phase**	***k***_**cat**_**(s**^**−1**^**)**	***K***_**m**_^**NADPH**^**(µM)**	***k***_**cat**_**/*****K***_**m**_**(M**^**−1**^**s**^**−1**^**)**
*wt*Fno	Phase 1	4.16±0.07^a^	2.3±0.2^a^	1.8×10^6^±1.6×10^5^^a^
I135V		1.50±0.09	0.7±0.1	2.1×10^6^±3.4×105
I135A		0.91±0.04	0.27±0.01	3.4×10^6^±1.9×10^5^
I135G		0.11±0.08	16±3	6.8×10^3^±5.1×10^3^
*wt*Fno	Phase 2	5.41±0.04^a^	62±6^a^	8.8×10^4^±8.4×10^3^^a^
I135V		2.16±0.02	51±7	4.2×10^4^±5.8×10^3^
I135A		1.24±0.02	2.9±0.3	4.2×10^5^±4.5×10^4^
I135G		0.33±0.19	654±100	5.0×10^2^±1.7×10^2^

a Values obtained from reference 16.

### 3.3 Pre steady-state kinetics of I135 Fno variants

The pre steady-state experiments for the Fno variants were conducted under the same conditions as *wt*Fno [16]. The reduction of the FO peak was monitored by absorbance changes at 420 nm. Like *wt*Fno, the spectra collected at different time intervals within the wavelength range of 350–800 nm did not show any formation of new peaks during the reaction, suggesting the absence of any observable intermediate peaks (Fig. S4) [16]. Also, the reaction progress curve exhibited an initial burst phase, followed by a slow phase, as shown in Fig. 5 (Fig. S5 displays the *wt*Fno trace) [16]. The burst suggests that the rate-limiting step is after the hydride transfer step. The data revealed that as the length of the side chain decreases (I135V, I135A and I135G Fno, respectively) there is an increase in the fast phase rate constant (Table 4). However, the side chain length has no effect on the rate constant of the slow phase (Table 4). The amplitude of the burst phase was directly proportional to the Fno concentration. Hence, this relationship allowed us to calculate the percentage of the site reactivity of Fno. The percentage of the site reactivity for *wt*Fno, I135V, I135A and I135G Fno are 54%, 64%, 44% and 51% respectively. This supports the half-sites reactivity model as reported previously for *wt*Fno [16]. A detailed description of the calculations for the Fno variants pre steady-state kinetics parameters is given in the supplemental information.

**Table 4:** Pre-steady state kinetics parameters of *wt*Fno and I135 variants. The rapid kinetic experiments were performed in the Hitech Scientific DX2 stopped-flow spectrophotometer at 22 °C. Fno (1.0, 1.5 and 2.0 μΜ; 50 mM MES/NaOH, pH 6.5, respectively) was mixed with 10 μM NADPH, forming the Fno-NADPH complex. FO (25 μM) in 50 mM MES/NaOH, pH 6.5 was then mixed with the Fno-NADPH complex. The calculation of the rate constants (*k*) of the slow phases along with the half-site reactivity is shown in the supplemental information.

**Fno**	**Burst phase*****k*****(s**^**−1**^**)**	**Slow phase*****k*****(s**^**−1**^**)**	**Half-site reactivity (%)**
*wt*Fno	47.9±0.5^a^	1.99±0.02^a^	54±1^a^
I135V Fno	293±22	2.34±0.01	64±2
I135A Fno	321±18	2.12±0.02	44±2
I135G Fno	1697±86	1.92±0.01	51±1

a values obtained from reference 16.

## 4 Discussion

Here, we examine the effects of I135 variants on the donor-acceptor distance between FO and NADPH using binding studies, steady-state and pre steady-state kinetic methods. The conversion of I135 to a valine, alanine and glycine decreased the length of the side chain by one carbon atom, respectively. Our results were compared to *wt*Fno, which was reported previously [16].

Our binding studies suggest that I135 plays a minimal role in the binding of both NADPH and FO. Decreasing the length of the side chain at residue 135 causes Fno to have decreased affinity for FO (up to a 2 fold increase in the *K*_d_ values (see Table 1). However, the enzyme does seem to adjust to losing side chain length, given that the binding affinity remains in the low nM range with each variant in comparison to *wt*Fno. The data displayed increased NADPH dissociation constants with respect to the I135V and I135A Fno variants. However, the I135G Fno variant revealed a similar *K*_d_^NADPH^ to that of *wt*Fno [16]. The Hill coefficients for *wt*Fno, along with the Fno variants are all less than 1. This supports the observation of negative cooperativity kinetics for *wt*Fno, as well as the I135 Fno variants. However, the Hill coefficient for the variants display higher values than that of the *wt*Fno, which suggests decreased negative cooperativity for the variants in comparison to *wt*Fno.

The steady-state Fno data yielded typical Michaelis-Menten plots with *k*_cat_ values, in reference to FO, that decreased with decreasing side chain length. The same phenomenon is shown with the catalytic efficiency (*k*_cat_ /*K*_m_) of the enzymes (Table 2). However, *K*_*m*_^FO^ remained unaffected. The *K*_d_^FO^ values are in the nM range, while the *K*_m_^FO^ values are in the μM range. The difference in the magnitude of these parameters could be due to the different experimental conditions. The dissociation constants were determined using Fno and FO, or Fno and NADPH, but not both simultaneously. Therefore, there was no turnover. The Michaelis-Menten constants were determined during catalysis in the presence of both FO and NADPH, which affects *k*_on_, *k*_off_, as well as *k*_cat_. These three terms affect the magnitude of *K*_m_. However, the *K*_d_ values are only affected by *k*_on_ and *k*_off_ [16].

The steady-state kinetic data for the Fno variants with respect to NADPH revealed plots that do not display Michaelis-Menten kinetics. This observation is consistent with our previously published data with *wt*Fno [16]. The NADPH steady-state kinetic data are biphasic, for all of the variants, with double reciprocal plots that displayed a downward concave shape, indicative of negative cooperativity (Fig. 4). Typically, when the value of the Hill coefficients are less than one, this is indicative of negative cooperativity binding. Our kinetic data is consistent with the Hill coefficients of *wt*Fno, along with the variants, which are all less than 1 (Table 1). The *k*_cat_ for each phase decreased with decreasing side chain length. However, there is no observable trend with respect to the *K*_m_^NADPH^ or *k*_cat_/*K*_m_^NADPH^ of the I135 Fno variants.

In order to determine that the fast phase is a true burst and not an anomaly due to cofactor binding, we conducted a pre steady-state experiment with NADP^+^ and FO cofactor under similar conditions as described previously under the pre steady-state methods section. Based upon our previous studies of *wt*Fno with NADP^+^ and FO cofactor [16], as well as the Fno crystal structure [1], it is clear that both oxidized cofactors bind Fno. Our pre steady-state experiment shows a straight line with no change in absorbance over a 30 s time period. The biphasic kinetics is not observed with cofactor binding and is only observed during catalysis (see supporting information Fig. S6).

**Fig. 6:**
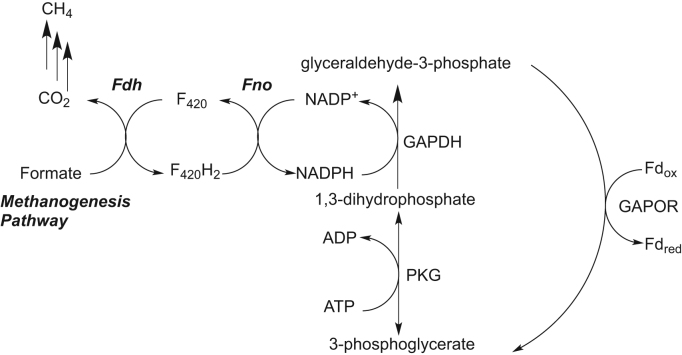
Diagram showing possible connection between methanogenic intermediates and glycolytic intermediates within Fno producing cells. The glycolytic intermediates are shown with potential input from F_420_H_2_ and NADPH produced from Fno [19]. Enzymes connecting the pathways include: Fdh, formate dehydrogenase, glyceraldehyde-3-phosphate (G3P): ferredoxin oxidoreductase (GAPOR), G3P dehydrogenase (GAPDH), and phosphoglycerate kinase (PKG), an ATP-dependent enzyme [19].

The pre steady-state data with F_420_ cofactor and NADPH for the Fno variants revealed biphasic kinetics with a fast and slow phase, similar to what was seen previously with *wt*Fno [16]. Fig. S7 displays the same data set at longer times to show that the slow phase continues past 1 min. The I135 variants showed an increase in the rate constant of the fast phase as the length of side chain decreased (Table 4). This increase in burst phase rate constant reflects an increasing rate of hydride transfer. The crystal structure by Warkentin et. al. shows the carbonyl oxygen of the I135 residue within 3.1 Å of the C4 of NADP^+^(Fig. 2b) [1]. They suggest that this distance could have catalytic relevance (Fig. 2). I135 is connected to a loop that is within 3.0 Å of NADP^+^. We suggest that this loop is flexible and undergoes conformational changes within the enzyme due to successive loss of carbon atoms with each variant. Our suggestion is that the enzyme reorganizes to bring the two cofactors within close proximity, thereby increasing the rate of hydride transfer. It appears that the I135 side chain aids in regulating the rate at which NADPH is produced within the cell. While the variants display faster hydride transfer than *wt*Fno, clearly, the cell doesn’t require a faster rate of hydride transfer such as what is observed in the I135 variants and therefore, utilizes an isoleucine residue for steady NADPH production. NADPH production is likely dependent upon the cell's need for this molecule as well as gluconeogenic/glycolytic intermediates [19]. *Leigh et. al* suggest that the NADPH produced by Fno is used for the production of glyceraldehyde-3-phosphate, 3-phosphoglycerate as well as the re-oxidation of the F_420_ cofactor that can be regenerated by Fdh for methanogenesis (Fig. 6) [19].

The rate constant for the second phase appears unaffected for all of the variants. These variants, like *wt*Fno [16] also display half-site reactivity in which only half of the cofactor is reduced (Table 4). The data suggest that this step is rate-limiting in catalysis. Given that burst kinetics is observed within Fno, it is plausible that the slow step could be product release or conformational changes within Fno.

In summary, we have applied binding studies, steady-state and pre steady-state kinetic methods to assess the kinetic behavior of I135, along with its effects on the donor-acceptor distance between FO and NADPH. The steady-state and pre steady-state kinetics of the I135 Fno variants suggests that I135 modulates the donor-acceptor distance between the two cofactors, which affects the rate at which the hydride is transferred (fast phase) within Fno. I135 also affects *k*_cat_ and has a minimal effect on FO binding. Fno is a dynamic and regulatory enzyme that modulates NADPH production within the cell.
