# Supplementary material for: Effects of isoleucine 135 side chain length on the cofactor donor-acceptor distance within F420H2:NADP+ oxidoreductase: A kinetic analysis
Source: Biochem Biophys Rep. 2016 Nov 30;9:114–20. doi: 10.1016/j.bbrep.2016.11.012 (PMC5614548; doi:10.1016/j.bbrep.2016.11.012)
Supplement: Supplementary file 1 — Supplementary material [file mmc1.docx]

There's no financial/personal interest or belief that could affect objectivity.

Our funding source is through the NIH Grant 1 R15 GM113223-01A (to KJW).
