# Supplementary material for: Effects of isoleucine 135 side chain length on the cofactor donor-acceptor distance within F420H2:NADP+ oxidoreductase: A kinetic analysis
Source: Biochem Biophys Rep. 2016 Nov 30;9:114–20. doi: 10.1016/j.bbrep.2016.11.012 (PMC5614548; doi:10.1016/j.bbrep.2016.11.012)
Supplement: Supplementary file 2 — Supplementary material [file mmc2.docx]

**Effects of Isoleucine 135 side chain length on the cofactor donor-acceptor distance within F_420_H_2_:NADP^+^ Oxidoreductase: A kinetic analysis**

**Cuong Quang Le^1^, Mercy Oyugi^2^, Ebenezer Joseph, Toan Nguyen, Md Hasmat Ullah, Joshua Aubert, Thien Phan, Joseph Tran and Kayunta Johnson-Winters***

Department of Chemistry and Biochemistry, University of Texas at Arlington, Texas 76019-0065

*Phone: 817-272-3802, Fax: 817-272-3808, Email: [kayunta@uta.edu](mailto:kayunta@uta.edu)

RECEIVED DATE (to be automatically inserted after your manuscript is accepted if required according to the journal that you are submitting your paper to)

1 and 2 contributed equally to this manuscript

† This research was supported by NIH Grant 1 R15 GM113223-01A (to KJW)

Running Title: F_420_ cofactor Dependent Enzymes

Table of contents

Page 3 Table S1. I135 Primers used for Fno site directed mutagenesis studies

Page 4-5 Figure S1. The binding of NADPH and FO to *wt*Fno and I135 variants

Page 6 Figure S2. The steady-state kinetics of *wt*Fno and the three I135 variants with varying [FO]

Page 7 Figure S3. The steady-state kinetics of *wt*Fno and the three I135 variants with varying [NADPH]]

Page 8 Calculation of the pre-steady state kinetic parameters

Page 9 Figure S4. The pre-steady state spectral evolutions of *wt*Fno and the Fno I135 variants.

Page 10 Figure S5. The absorbance (420 nm) versus time traces for the *wt*Fno and I135 Fno variants.

Page 11 Figure S6 Pre-steady state experiment with oxidized F_420_ and NADP^+^

Page 12 Figure S7 Pre steady-state data at a longer time point

| **Fno primers** | **Sequence (5’ to 3’)** |
| --- | --- |
| *wt*Fno | GCC CTG CAC ACG **ATC** CCG GCA GCT CGT TTT |
| I135A (F) | GCC CTG CAC ACG **GCC** CCG GCA GCT CGT TTT |
| I135A (R) | AAA ACG AGC TGC CGG **GGC** CGT GTG CAG GGC |
| I135G (F) | GCC CTG CAC ACG **GGC** CCG GCA GCT CGT TTT |
| I135G (R) | AAA ACG AGC TGC CGG **GCC** CGT GTG CAG GGC |
| I135V (F) | GCC CTG CAC ACG **GTC** CCG GCA GCT CGT TTT |
| I135V (R) | AAA ACG AGC TGC CGG **GAC** CGT GTG CAG GGC |

### Table S1. A list of the Fno I135 primers used for Fno site directed mutagenesis studies. The forward and the reverse primers were used to convert the active-site I135 isoleucine (I135) into alanine, glycine and valine residues, respectively.

**
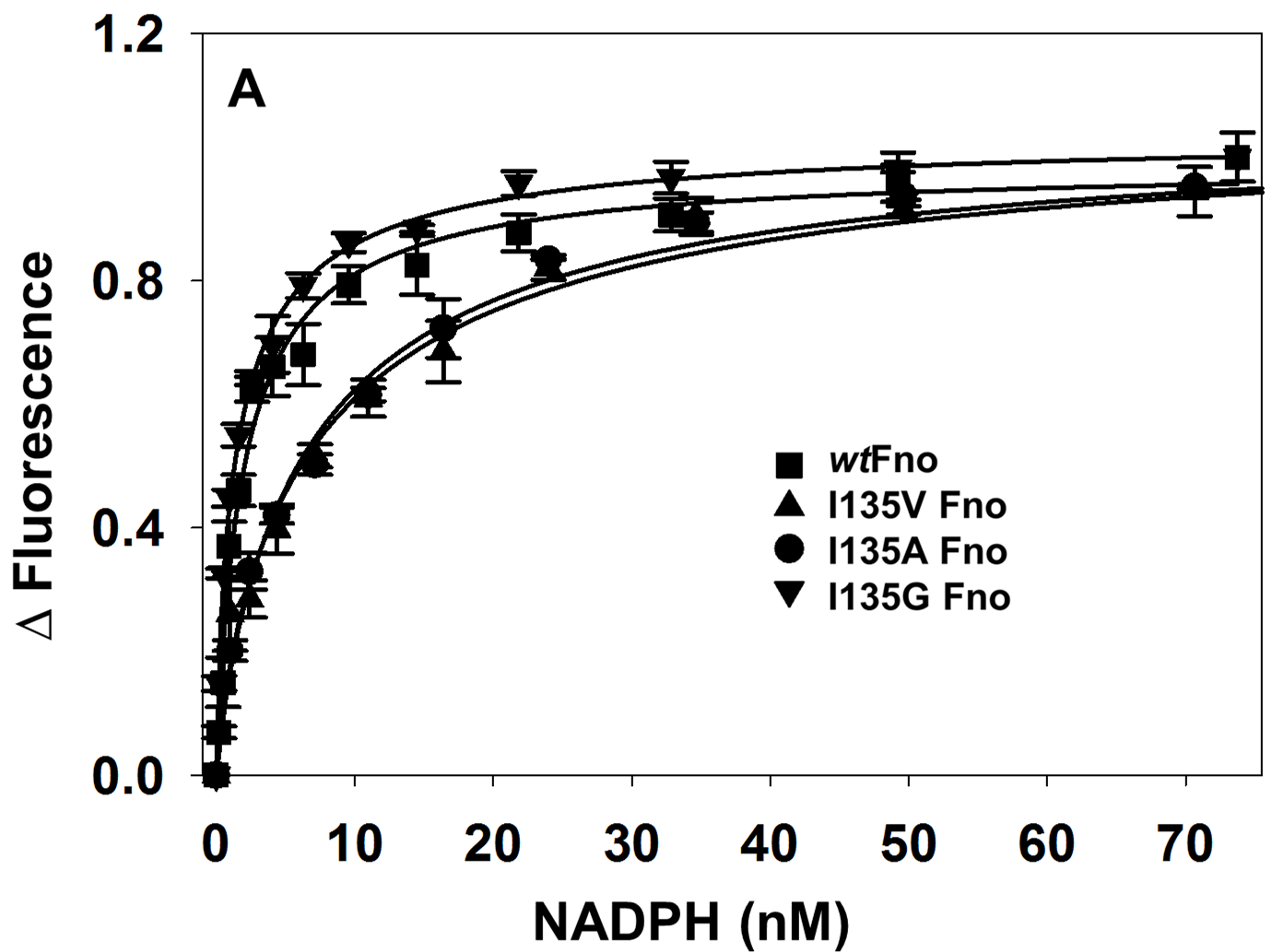
**

**
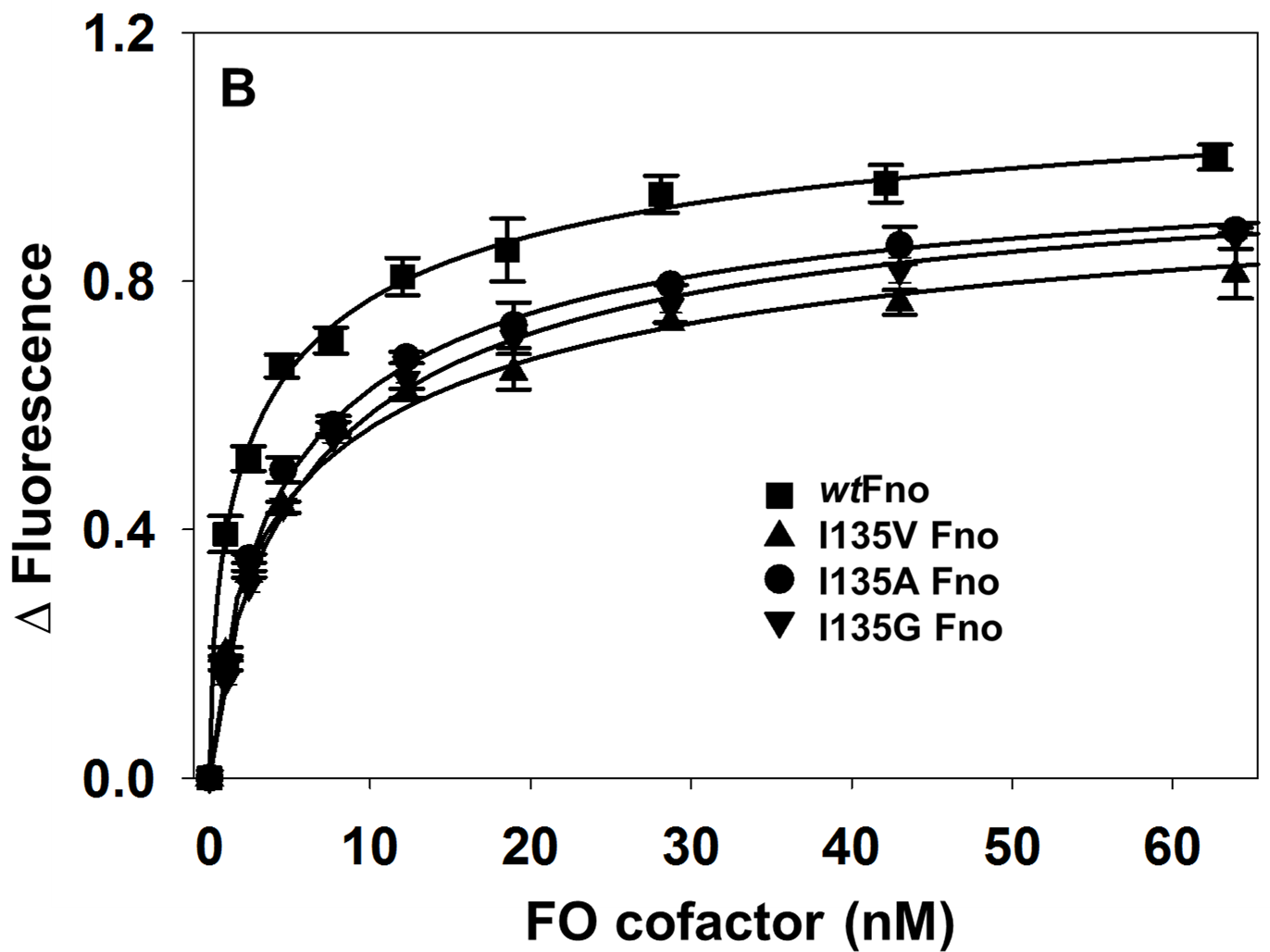
**

**Figure S1**. Fno binding. **A.** Plots of Δfluorescence vs. [NADPH] are displayed for *wt*Fno and I135 Fno variants. The experiments were carried out in 50 mM MES/NaOH (pH 6.5) buffer at 22 ^0^C in the Fluorometer. NADPH was titrated into 0.2 µM of Fno and the fluorescence emission was monitored at 340 nm after excitation at 290 nm. The actual fluorescence values were inverted and normalized prior to plotting. The solid lines represent fit to equation 1. **B.** Plots of Δfluorescence vs. [FO] are displayed. The experiments were carried out under similar conditions as described for NADPH binding and then fit to Equation 1, ΔF = [F_max_ [L]*^n^*/(*K_d_* + [L]*^n^*)].


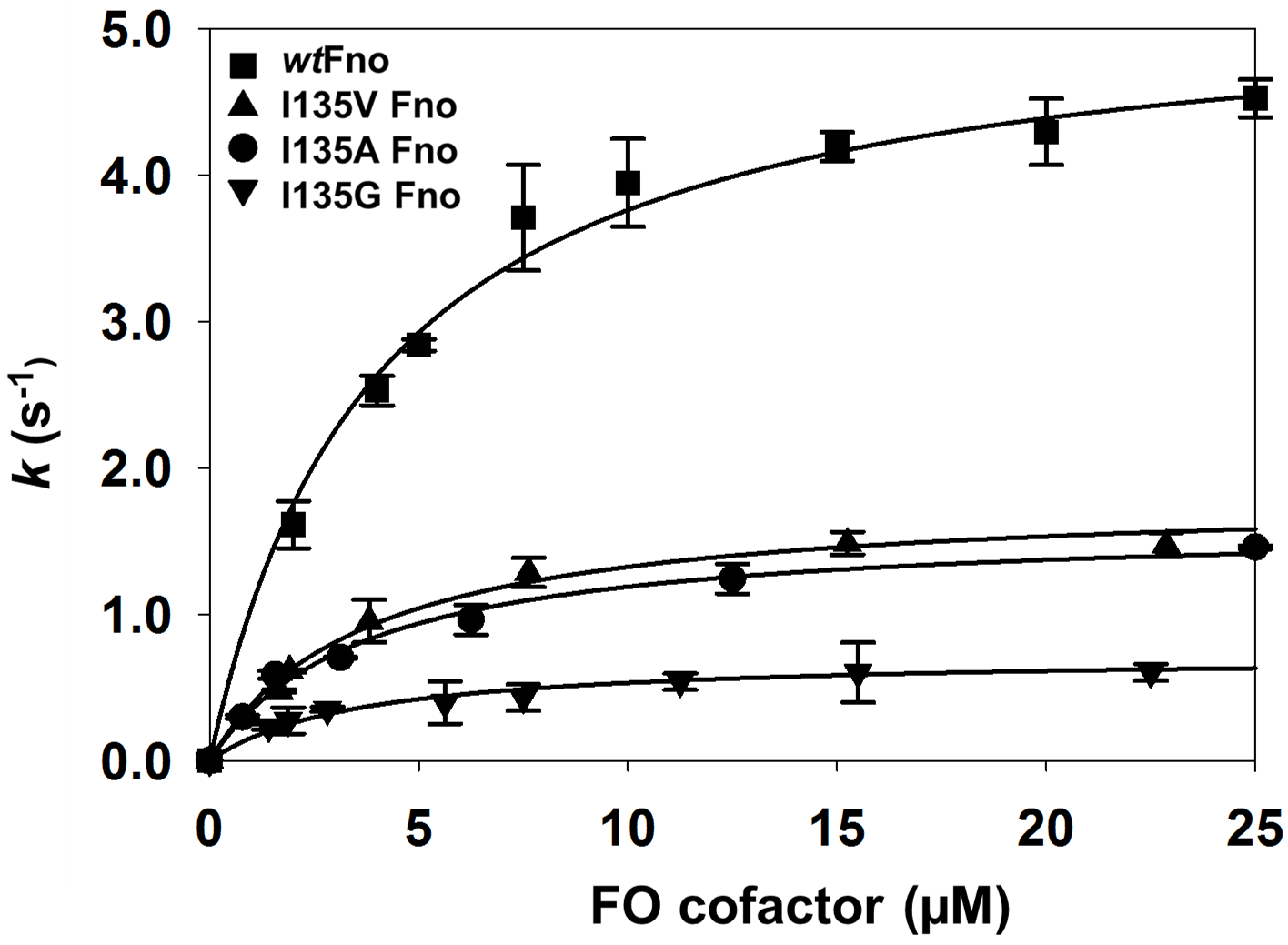


**Figure S2.** The steady state kinetic plots of *wt*Fno (0.2 µM) and the I135 Fno variants (0.2 µM) at varying FO concentrations. The reaction is carried out using 600 µM NADPH in 50 mM MES/NaOH (pH 6.5) buffer at 22 °C.

**Calculation of steady-state kinetic parameters with respect to varied [NADPH]**

To determine the kinetic parameters, we used SigmaPlot 13.0 version to fit the data to Equation S1 to determine the kinetic parameters of each phase of the Fno plots with respect to the [NADPH]. *k* is the first order macroscopic rate constant obtained by dividing the initial rate by the enzyme concentration; *K_m1_* and *K_m2_* are the Michaelis constants of the first and second phases of the plots, respectively; *k_1_* and *k_2_* are the rate constant for the first and second phases of the plot, respectively (See Figure S3).


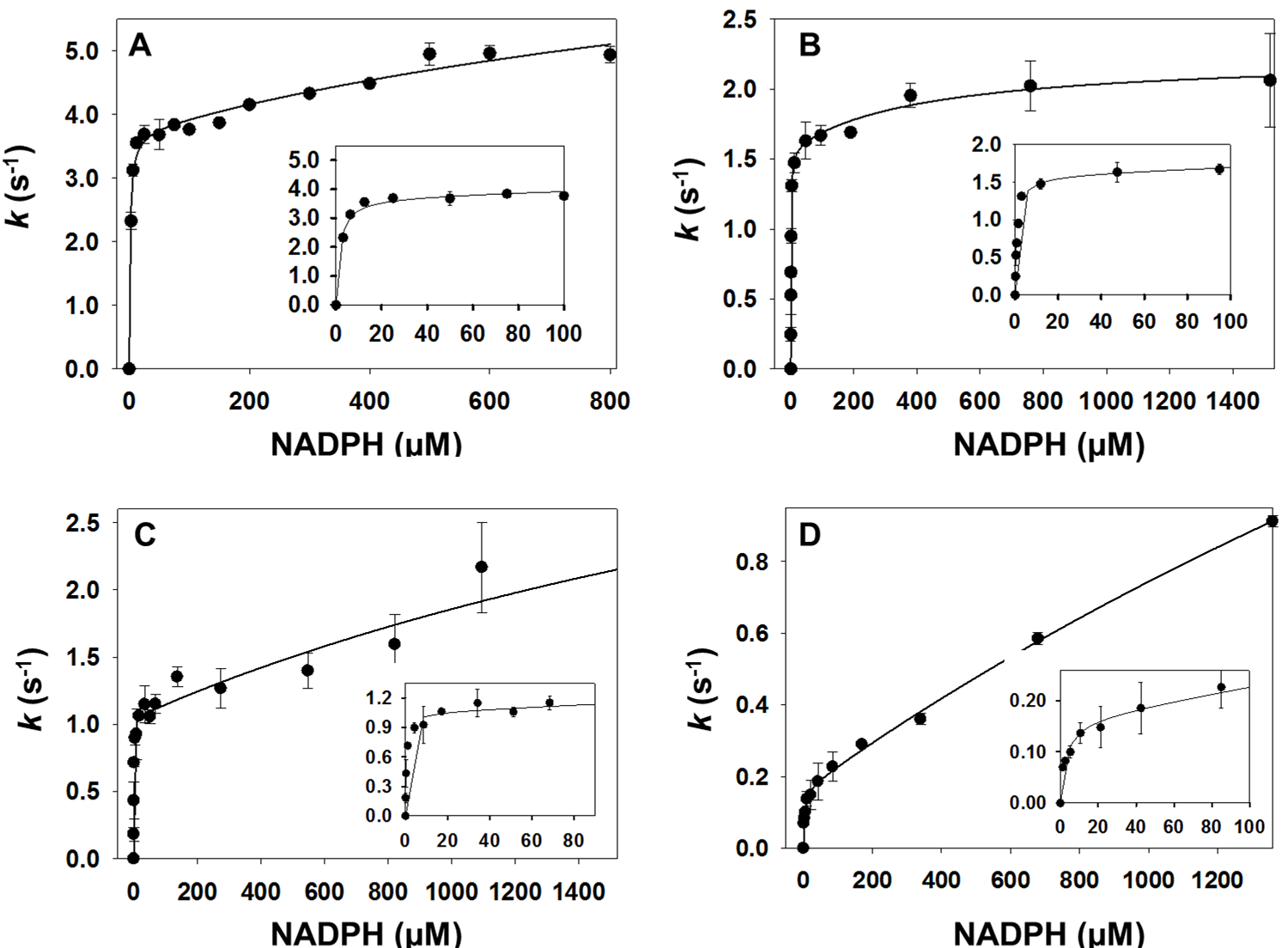


**Figure S3.** Biphasic steady state kinetic plots of *wt*Fno (**A)**, I135V Fno **(B),** I135A Fno **(C)** and I135G Fno **(D)** with varying [NADPH]. The figure represents plots of *k* versus [NADPH]. The reaction conditions consisted of Fno (0.2 µM, either *wt*Fno or Fno variants). The FO concentration was 25 μM. The sample was titrated with varying NADPH concentrations in 50 mM MES/NaOH (pH 6.5) buffer at 22 °C.

Equation S1

**Calculation of the pre-steady state kinetic parameters**

To determine the kinetic parameters for each Fno variants, we used SigmaPlot 13.0 version to fit our pre-steady state data to the exponential decay (Equation S2). The rate constant of the burst phase is *k* in Equation S2. Notice: *k* is already in units of per second based on the unit calculation. We obtained the slow-phase rate constant (*k*_s_) by dividing the slow-phase rate (*v_s_*) by the total Fno concentration in the reaction sample, after converting *v*_s_ into the unit of µM s^-1^ using the FO coefficient (Equation S3). For the Fno half-site reactivity, we divided the amplitude (A_o_) of the burst phase by the total Fno concentration ([E]_tot_) after converting the A_o_ into the unit of µM s^-1^ (Equation S4). This calculation was repeated to obtain all parameters of three different enzyme concentrations for each of the three Fno variants. The reported parameters in Table 4 are the average values.

Equation S2 A_420nm_ = A_o_e^(-^*^k^*^t)^ – *v*t + c

- A_420nm_ = absorbance at 420nm
- t = time (s)
- A_0_ = amplitude of the burst phase (unit is Abs)
- *k* = *k*_b_ = observed burst rate constant (s^-1^)
- *v* = *v*_s_ = observed slow-phase rate (Abs per second)
- c = nonzero baseline constant (Abs)

Equation S3

- *k*_s_ = slow-phase rate constant (s^-1^)
- *v*_s_ = slow-phase rate (Abs per second)
- ε = coefficient of FO cofactor (0.0347 uM^-1^cm^-1^; pH 6.5 at 420 nm)
- [E]_tot_ = total enzyme in a reaction sample (µM)

Equation S4

- H = half-site reactivity (%)
- ε = coefficient of FO cofactor (0.0347 uM^-1^cm^-1^; pH 6.5 at 420 nm)
- [E]_tot_ = total enzyme in a reaction sample (µM)
- A_o_ = amplitude of the burst phase (unit abs)
- [E]_tot_ = total enzyme in a reaction sample (µM)


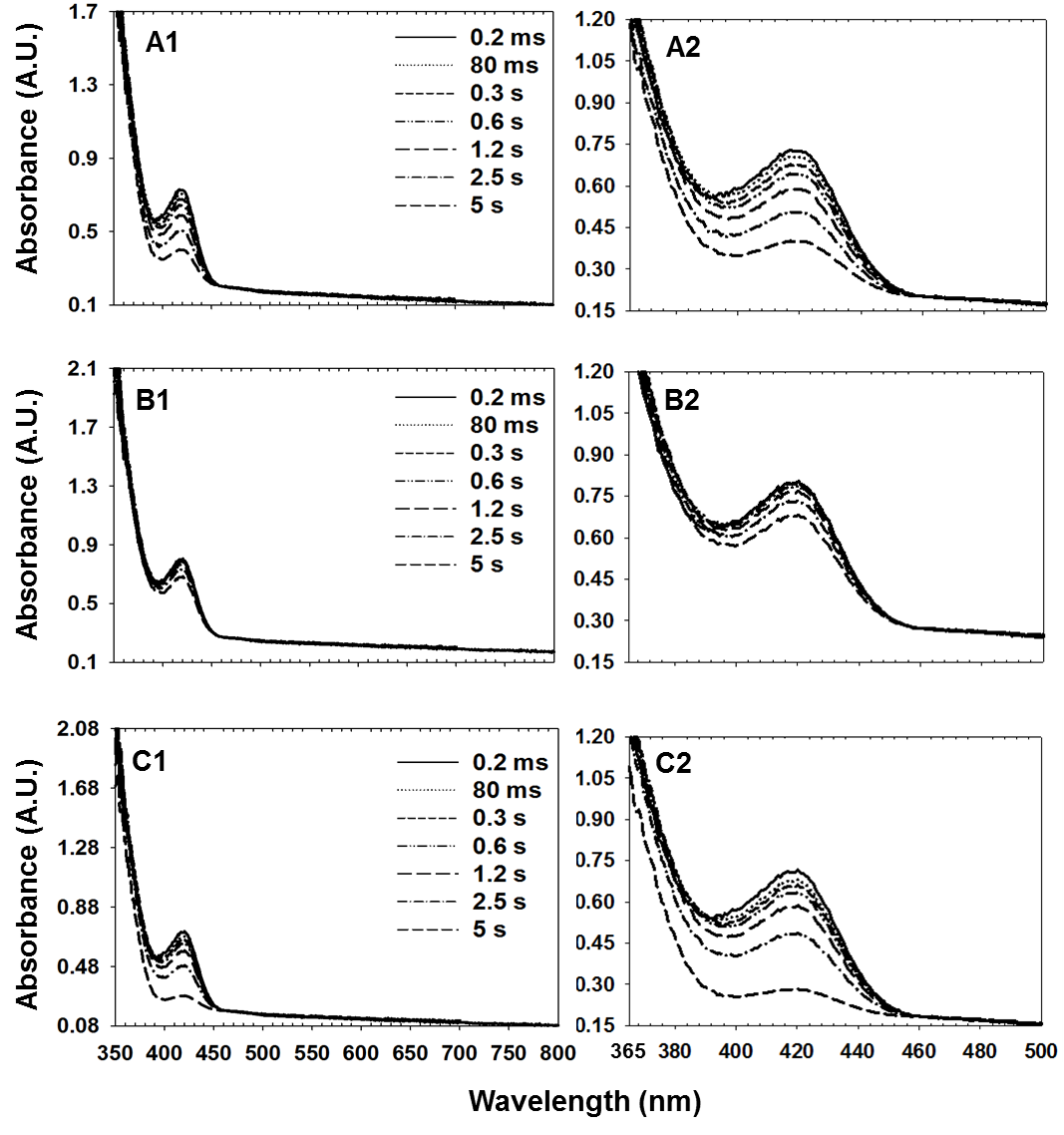


**Figure S4.** The pre-steady state spectral evolutions of *wt*Fno and the Fno I135 variants. The spectra were captured between 300 nm and 800 nm during the first five seconds of the reaction. Figure: A1) 2 µM I135V Fno B1) 2 µM I135A Fno and C1) 2 µM I135G Fno. Figure A2, B2 and C2 are expanded views of Figure A1, B1 and C1 from 365 to 500 nm, respectively. Notice that only a single peak at 420 nm was observed for all *wt*Fno, as well as the three I135 variants. The data showed decreased absorbance as time progressed (0.2 millisecond to 5 seconds). Hence, there was no intermediate observed during the pre-steady state reactions of Fno variants based on these spectra. The reactions were carried out in 50 mM MES/NaOH (pH 6.5) buffer at 22 °C. Fno was mixed with 10 μM NADPH, forming the Fno-NADPH complex. FO (25 μM) in 50 mM MES/NaOH, pH 6.5 was then mixed with the Fno-NADPH complex.

**
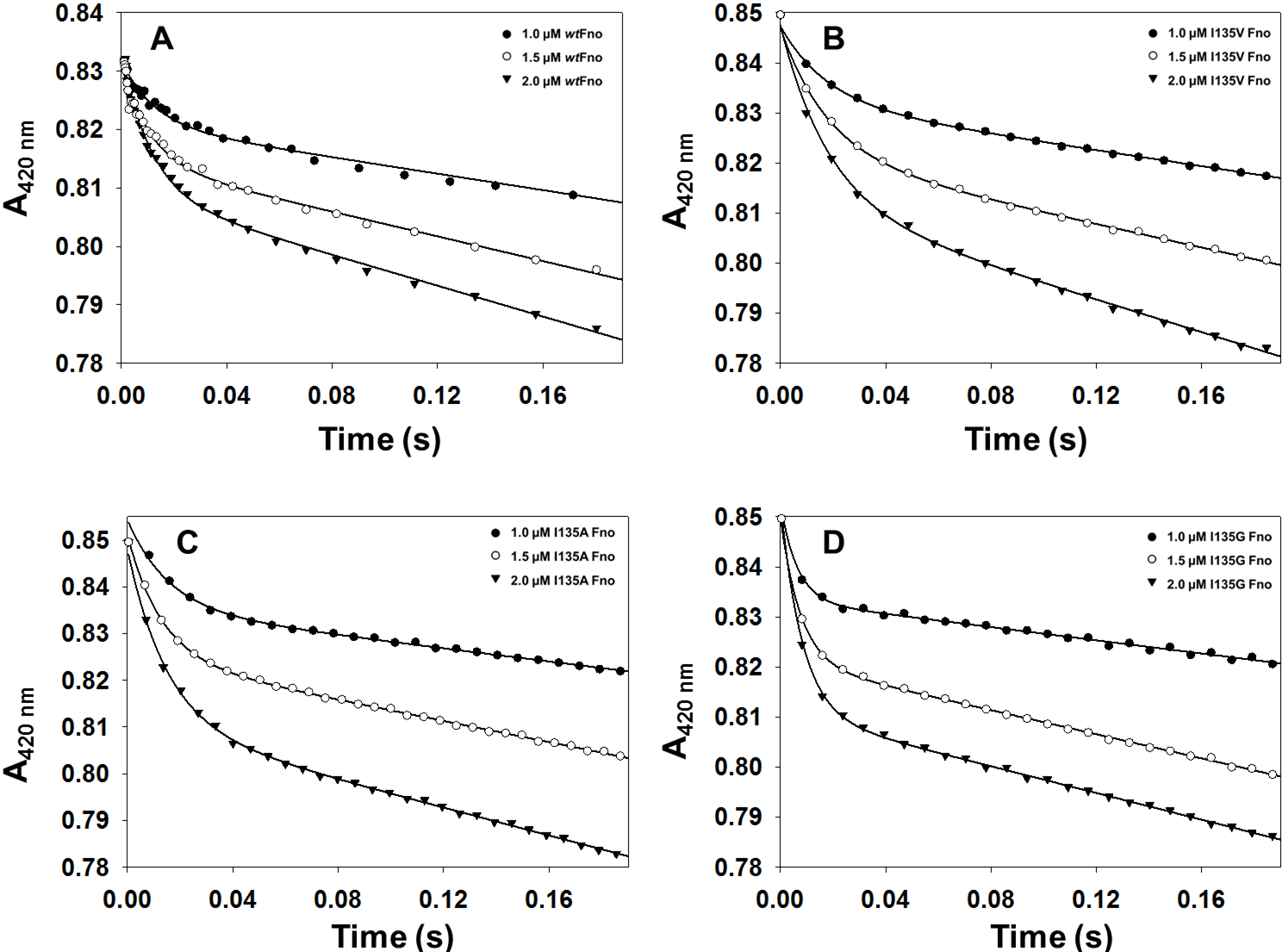
**

**Figure S5.** The absorbance (420 nm) versus time (s) traces at various enzyme concentrations for *wt*Fno (A), I135V Fno (B), I135A Fno (C) and I135G Fno (D).

Fno concentrations: 1.0 μM Fno (solid circles), 1.5 μM Fno (open circles), and 2.0 μM Fno (solid triangles). – The plots were fitted to Equation 5 and represent the three Fno variants as follows: A (I135V Fno), B (I135A Fno), and C (I135G Fno). The reactions were carried out in 50 mM MES/NaOH (pH 6.5) buffer at 22 °C. Fno was mixed with 10 μM NADPH, forming the Fno-NADPH complex. FO (25 μM) in 50 mM MES/NaOH, pH 6.5 was then mixed with the Fno-NADPH complex.


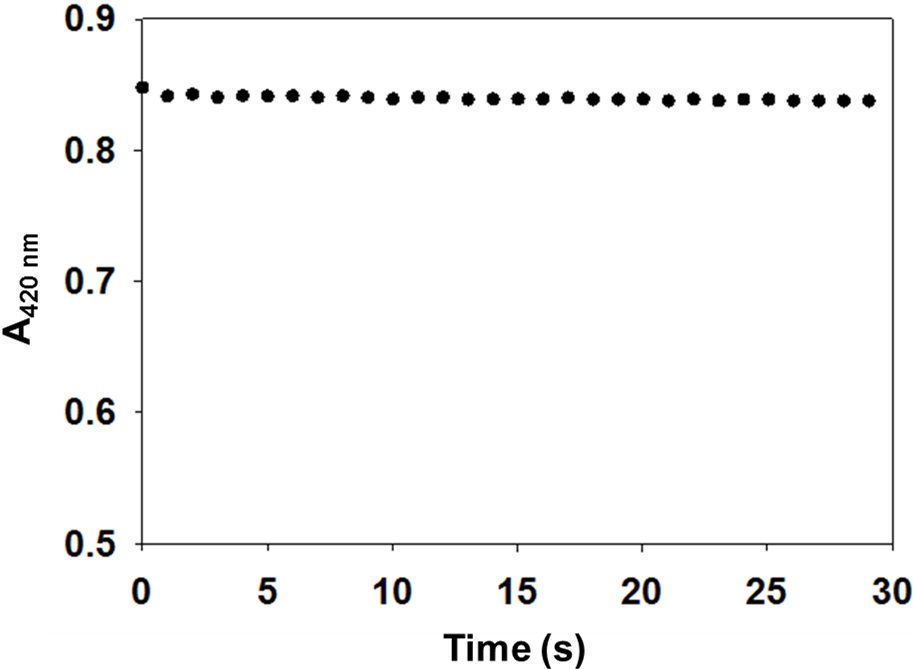


Figure S6: Pre-steady state experiment: The reaction was carried out in 50 mM MES/NaOH (pH 6.5) at 22 °C. Fno was mixed with 10 µM NADP^+^, forming the Fno-NADP complex. 25 µM FO in 50 mM MES/NaOH, pH 6.5 was then mixed with the Fno-NADPH complex. This pre-steady state data yielded a horizontal line, which indicates no enzymatic reaction. The data did not fit to any of the equations used previously to fit the pre steady-state data.


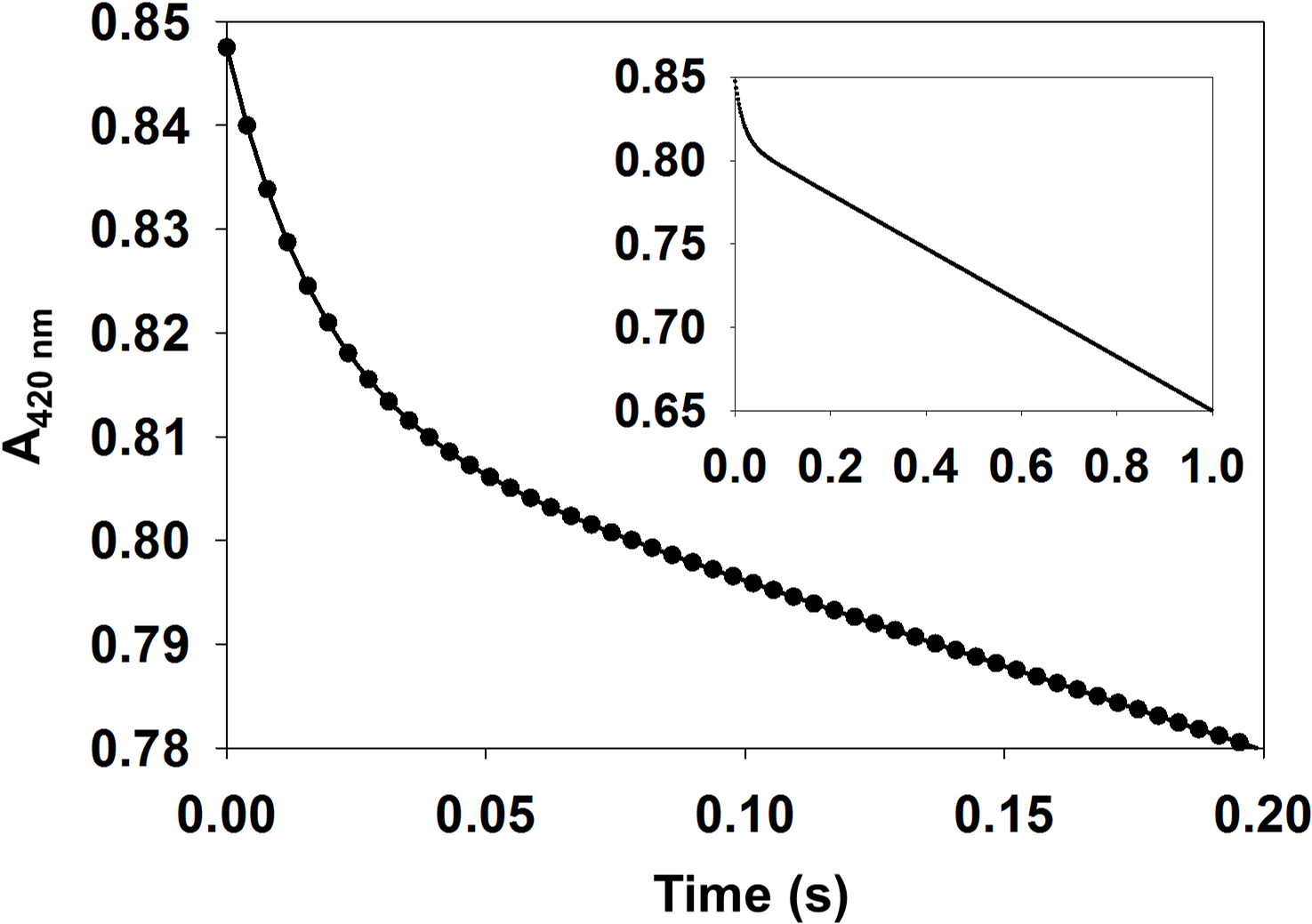


**Figure S7**. A representative curve of a longer time pre-steady-state for I135V Fno at a 2.0 µM concentration. The plots were fitted to Equation 5. The reaction was carried out in 50 mM MES/NaOH (pH 6.5) at 22 °C. Fno was mixed with 10 µM NADPH, forming the Fno-NADPH complex. 25 µM FO in 50 mM MES/NaOH, pH 6.5 was then mixed with the Fno-NADPH complex.
